# Geochemical Characteristics and Environmental Implications of Surface Sediments from Different Types of Sand Dunes in the Dinggye Area, Southern Tibet

**DOI:** 10.3390/ijerph191710628

**Published:** 2022-08-26

**Authors:** Meihui Pan, Yougui Chen, Zewen Hao, Chenlu Li, Huimin Zhao, Jinyu Wang, Yifu Gong

**Affiliations:** 1 College of Geography and Environment Science, Northwest Normal University, Lanzhou 730070, China; 2 Key Laboratory of Resource Environment and Sustainable Development of Oasis, Gansu Province, Lanzhou 730070, China

**Keywords:** Dinggye area, sand dune, geochemical characteristics, provenance, multidimensional scaling, principal component analysis

## Abstract

Geochemical characteristics of aeolian sand are beneficial for understanding sand dune formation and evolution. Few studies in the Dinggye area, Southern Tibet, have focused on the geochemical characteristics of aeolian sand. Thus, we present new geochemical data that provide insights into the geochemical characteristics and environmental implications of aeolian sands in the Dinggye area. The results show that mobile dunes, climbing sand sheets, and nebkhas show heterogeneity in elemental concentrations and UCC-normalized distribution; MgO, TiO_2_, Ni, Pb, and Nb are higher in mobile dunes; SiO_2_, CaO, K_2_O, Na_2_O, P_2_O_5_, V, Cr, Co, Cu, Ba, and Ce are higher in climbing sand sheets; and Al_2_O_3_, Fe_2_O_3_, La, Zn, As, Sr, Y, Zr, Rb, and Ga are higher in nebkhas. Principal component analysis (PCA) and correlation analysis indicate that the main factor affecting elemental content is grain size sorting, followed by provenance, while chemical weathering and regional precipitation are less influential. The CIA and A-CN-K triangle indicate that the different dune types are at a lower chemical weathering stage, with plagioclase weathering and decomposition first. The combination of grain size characteristics, elemental ratios, multidimensional scale (MDS), PCA, and geomorphological conditions suggest that the flood plain and the lakeshore are the main sand sources of aeolian sands in the Dinggye area.

## 1. Introduction

Aeolian landforms are an important part of the sedimentary system on the Earth’s surface, mainly in arid and semi-arid areas [1,2], and their formation and evolution have a strong influence on the local climate, air quality, and inhabitants [3,4]. Aeolian landforms have evolved over more than 100 years, and they have made great strides in terms of dune morphological features and dynamics [5]. In recent years, the study of aeolian activities and landforms has become an important aspect of global change research, as aeolian hazards have become more prevalent [6,7]. Many studies have been conducted on the geochemical characteristics of sands, and the results have contributed significant insights into climate change, land surface processes, and Earth systems [8,9,10].

Geochemical characteristics are the main components in the study of aeolian landform processes, which contain rich information on surface processes and climate change, and have great significance for provenance analysis, the recovery of climate zones, and the reconstruction of paleo-atmospheric circulation [11,12,13,14,15]. The characteristics of aeolian sand are crucial in controlling the processes of sand dune formation and evolution [16]. Foreign studies on the geochemical characteristics of aeolian landforms have used elemental ratios, the A-CN-K triangle, and discriminant analysis to analyze sand sources and environmental implications [17]. Many scholars have researched the geochemical characteristics of aeolian sediments on the Qinghai-Tibet Plateau (QTP) of China; these studies demonstrate the potential for geochemical methods to identify sediment sources and to understand surface processes in drylands [18,19,20,21]. However, previous studies have mostly emphasized single sand dunes, and there is limited research on the systematic comparison of different types of aeolian landforms.

The QTP is a unique alpine arid geographical unit that is covered with aeolian sediments [22]. The Dinggye region is located between the southern edge of the QTP and the northern edge of the Himalayas and is very sensitive to changes in local climatic conditions [23]. Aeolian landforms are widely distributed in this region, and their dynamic of formation is derived from the southern slope of the Himalayas, with a different mechanism of genesis from that of other regions of China [24]. Existing investigations on aeolian landforms in the Dinggye area have been analyzed mainly from the perspective of grain size and paleoclimate [25], but an understanding of geochemical characteristics is still lacking. This paper quantifies the geochemical characteristics and differences in the surface sediments of different types of sand dunes in the Dinggye area so as to identify their environmental importance, which can enrich the theory of desertification in the plateau area and also provide a scientific basis for land desertification control in the Dinggye area.

## 2. Materials and Methods

### 2.1. Study Area

**Figure 1:**
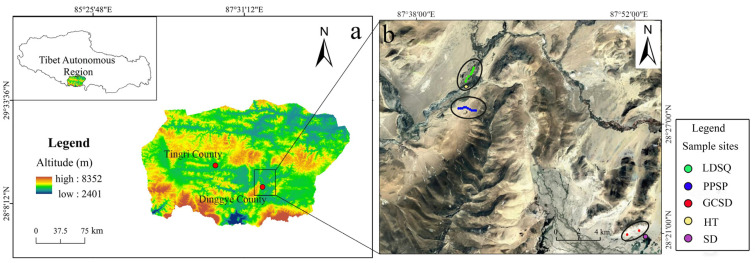
Location of the study area and sampling sites: (**a**) is the map of the Dinggye area and the Tibet Autonomous Region, and (**b**) is the location diagram of sampling points (image modified from Google Earth).

The Dinggye area (Figure 1) is located on the southern edge of the QTP [26]. The topography is dominated by high mountains, with river beaches and small plains with wide valleys of lake basins [27]. The stratigraphy in the study area shows an old to new distribution from south to north, with fault contacts between the strata. The Fourth Series is widely exposed in the study area. Alluvial deposits in the study area are mainly distributed along the valley areas of Pangqu, Jilong Zangbo, and Yeru Zangbo, mainly concentrated in the wide valley section. The intrusive rocks in the study area are more developed and mainly distributed in the central part of the study area. This region is influenced by the plateau temperate semi-arid climate [28], with an average annual temperature of 2 °C and the highest (12 °C) and lowest (−8 °C) temperatures occurring in July and January. The region receives 236 mm y^−1^ in rainfall, of which 70% of the falls are mainly concentrated in July and August, and the potential evaporation exceeds 3000 mm. The Dinggye area is mainly influenced by the combined effect of wind and river, the prevailing wind direction is mainly SSW, and the type of weathering is mainly physical weathering [29]. Vegetation coverage is relatively low (approximately 30–60%), with the main types including thickets, meadow, grasslands, alpine vegetation, and drought-tolerant crops [23]. Soils mainly include cryocalic, frost-calc soils, a small number of aquatic soils, frozen thin soils, and ordinary gleysols [28]. Climbing dunes, nebkhas, crescent dunes, flat dunes, and mobile dunes are the main aeolian landform types in the Dinggye area, and mainly along the Pengqu River, Xielin Zangbo, Yeru Zangbo, Jilong Zangbu, and Quqiang Zangbu valleys, they have a striped distribution and are concentrated in the wide valley section [24].

### 2.2. Sampling

**Figure 2:**
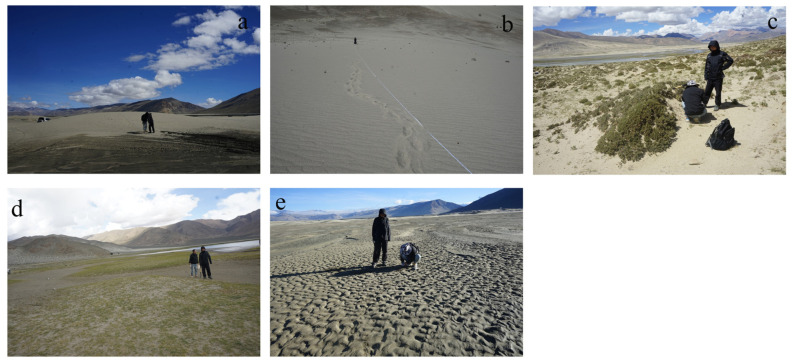
Map of sampling sites: (**a**) is mobile dune, (**b**) is climbing sand sheet, (**c**) is nebkha, (**d**) is lakeshore, and (**e**) is flood plain.

As in Figure 1 and Figure 2, a total of 154 samples were collected in the study area in September 2020. Samples were collected at 5 m intervals on the windward slopes of climbing sand sheets (PPSP1 and PPSP2), and a total of 35 and 18 samples were collected on PPSP1 and PPSP2 with slope lengths of 180 and 100 m, respectively. The seven mobile dunes had an average windward slope length of 16.31 m and an average leeward slope length of 4.8 m. The windward slope was sampled at 6 m intervals, and the leeward slope was sampled at three points (upper, middle, and lower), recorded as LDSQ1–LDSQ7. The 10 nebkha sites (GCSD1–GCSD10) had relatively high vegetation cover; three samples were collected from each of the dunes. Three samples were collected at equal intervals of 10 m along the lakeshore, between the wetland and the hillside mesas, which were wetter and dominated by sand (named SD1–SD3). Two samples were collected in the flood plain (named HT1–HT2). The sampling points were located using GPS. All of the above abbreviations apply in the following.

### 2.3. Laboratory Measurements and Methods

#### 2.3.1. Major and Trace Elements

Samples were air-dried at room temperature, then roots and impurities were removed and ground to <200 mesh using a vibrating grinding method. A total of 5 g of ground sample was weighed, and a boric acid fixed concentric cake specimen was made using a press with a pressure of 25 t and a holding time of 30 s, and the sample number was marked on the back. The compressed samples were measured using a PANalytical Epsilon 4 benchtop energy dispersive X-ray fluorescence spectrometer from PANalytical, The Netherlands. The major elements are given as percent oxides, and the trace elements are in ppm. The chemical index of alteration (CIA) value calculation formula [30] and the correction formula of CaO* [30,31]) are defined as:

(1) $$\;\;\;\;\;\;\;\;\;\;\;\;\;\;\;\;\;\;\;\;\;\;\;\;\mathrm{CIA}=\frac{{\mathrm{Al}}_{2}O_{3}}{{\mathrm{Al}}_{2}O_{3}+{\mathrm{Na}}_{2}O+{\mathrm{CaO}}^{*}+K_{2}O}\times 100$$

(2) $${\mathrm{CaO}}^{*}=\mathrm{CaO}-\left(\frac{10}{3}\times P_{2}O_{3}\right)$$

In these two equations, CaO* is the content of CaO stored in silicate minerals, excluding the content of CaO in carbonate and phosphate [30]. Due to CaO and Na_2_O in silicates usually existing in a 1:1 ratio, McLennan concluded that mCaO* = mNa_2_O when the molarity of CaO was greater than Na_2_O, and mCaO* = mCaO when it was less than or equal to Na_2_O. All mCaO* values in the paper are obtained according to this method [31].

#### 2.3.2. Grain Size

The experimental procedure for particle size analysis is divided into two parts: sample pretreatment and measurement; the pretreatment method is mainly based on Lu and An [32]. Calcium carbonate and organic matter are removed during the pretreatment process. The grain size of the samples was measured using the Malvern Matersizer 3000 laser grain size analyzer, which has a measuring range of 0.02~3500 μm. In the instrumental analysis, the degree of shading of the sample analysis is controlled from 10% to 30%, and samples with a degree of shading >30% and <10% need to be remeasured. The specific formula for the particle size parameter [33] is defined as:

(3) Φ=−log(D, 2)

(4) $$\mathrm{Mz}=\frac{\Phi 16+\Phi 50+\Phi 84}{3}$$

(5) $$\sigma =\frac{\Phi 84-\Phi 16}{4}+\frac{\Phi 95-\Phi 5}{6.6}$$

In the equations, Φ5, Φ16, Φ50, Φ84, and Φ95 are the grain sizes where the cumulative percentage reaches 5%, 16%, 50%, 84%, and 95%, respectively.

#### 2.3.3. Multidimensional Scaling

Multidimensional scaling (MDS) is commonly used to measure the similarity of multidimensional (usually two-dimensional) spatial data [34]. The points are repeatedly moved so that the fit between the distances and the data improves until no further improvement is possible. The higher the similarity between two objects, the smaller the distances in MDS space. The formula is as follows:

(6) $$\delta_{\mathrm{jk}}=\sqrt{\sum_{i=1}^{p}{\left(y_{\mathrm{ij}}-y_{\mathrm{ik}}\right)}^{2}}$$

where δ_jk_ is the dissimilarity between samples j; k and p refer to the number of properties used to perform MDS for every two objects, and y is fingerprint property (the elemental concentration for this study).

The best fit of an MDS solution can be measured using Kruskal Stress (also known as ‘Stress-1’) and the determination coefficient (RSQ). A Stress-1 value of <0.025 is perfect, <0.05 is excellent, <0.1 is good, <0.2 is fair, and >0.2 is poor [35]. RSQ is the proportion of total variance carried by the model that occupies the proportion of the total variance of the original data; an RSQ value of >0.6 is acceptable [35].

#### 2.3.4. Principal Component Analysis

Principal component analysis (PCA) is a statistical analysis method that reduces multiple indicators into a few composite indicators [36]. Using dimensionality reduction, it reduces multiple variables into a few principal components that retain most of the information of the original variables, finding some major components in the complex relationship of things, effectively using a large number of statistical data for quantitative analysis and revealing the relationship between variables [37].

## 3. Results

### 3.1. Major Element Composition

**Figure 3:**
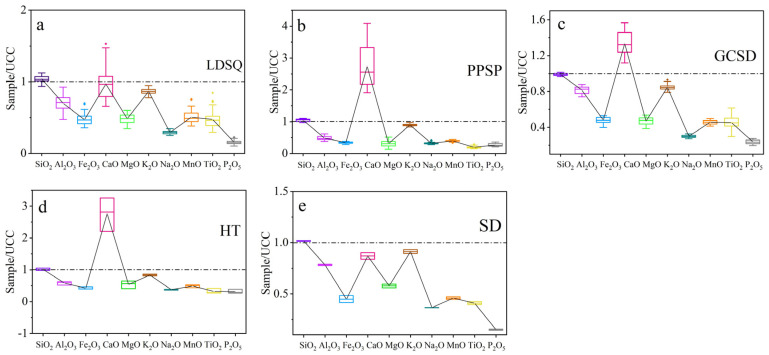
UCC-standardized box line diagram of trace elements for different types of sediments: (**a**) represents mobile dune, (**b**) represents climbing sand sheet, (**c**) represents nebkha, (**d**) represents flood plain, and (**e**) represents lakeshore. UCC datas are reprinted with permission from ref. [38]. 1985, Taylor and McLennan.

Major elemental datas for mobile dunes, climbing sand sheets, and nebkhas are presented in Table 1 and Figure 3. Overall, the major elements are dominated by SiO_2_, Al_2_O_3_, and CaO, with the sum of the three being >80%, which is absolutely dominant; Fe_2_O_3_, K_2_O, MgO, and Na_2_O are the next most abundant, while TiO_2_, P_2_O_5_, and MnO are less abundant, with an average content of <0.3%. Compared to UCC, SiO_2_ is essentially the same as UCC; CaO is enriched, and the remaining major elements are deficient. Mobile dunes comprise an average of 68.63% SiO_2_, 10.64% Al_2_O_3_, and 4.06% CaO; with 1.06% Fe_2_O_3_, 2.95% K_2_O, 1.06% MgO, and 1.14% Na_2_O and <0.30% of TiO_2_, P_2_O_5_, and MnO; compared with UCC, SiO_2_ and CaO are enriched, and the degree of deficit is P_2_O_5_ > Na_2_O > Fe_2_O_3_ > TiO_2_ > MgO > MnO > Al_2_O_3_ > K_2_O. The coefficient of variation (CV) is <18% except for TiO_2_, indicating a consistency in the composition of the major elements of mobile dunes. Climbing sand sheets comprise an average of 69.14% SiO_2_, 7.42% Al_2_O_3_, and 11.47% CaO, with 1.71% Fe_2_O_3_, 3.05% K_2_O, 0.69% MgO, and 1.26% Na_2_O, and <0.20% TiO_2_, P_2_O_5_, and MnO; SiO_2_ and CaO are enriched and the remaining elements are deficient as Ti_2_O > P_2_O_5_ > MgO > Na_2_O > Fe_2_O_3_ > MnO > Al_2_O_3_ > K_2_O. The CVs of CaO and MgO are >18%, and the remaining elements are <18%, indicating that there is also a good agreement in the major elements of the climbing sand sheets. The average contents of SiO_2_, Al_2_O_3_, CaO, Fe_2_O_3_, K_2_O, MgO, and Na_2_O in the nebkhas are 65.29%, 12.39%, 5.60%, 2.40%, 1.03%, 2.88%, and 1.17%; TiO_2_, P_2_O_5_, and MnO contents are 0.23%, 0.03%, and 0.12%; SiO_2_ is consistent with UCC, CaO is enriched and the remaining elements are deficient as P_2_O_5_ > Na_2_O > TiO_2_ > MnO > MgO > Fe_2_O_3_ > Al_2_O_3_ > K_2_O; the CVs for all major elements are <18%.

### 3.2. Trace Element Composition

**Figure 4:**
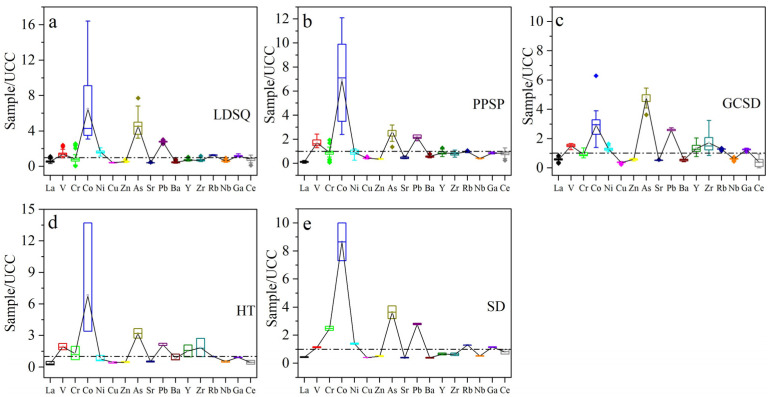
UCC-standardized box line diagram of trace elements for different types of sediments: (**a**) represents mobile dune, (**b**) represents climbing sand sheet, (**c**) represents nebkha, (**d**) represents flood plain, and (**e**) represents lakeshore.

Trace elemental datas for mobile dunes, climbing sand sheets, and nebkhas dune are presented in Table 2 and Figure 4. Dune surface sediments are dominated by Ba, Sr, Zr, and Rb, with an average content >100 ppm, followed by V, Co, and Ce, with lower levels of Cu, As, Y, Nb, and Ga. Relative to the UCC, V, Co, As, and Pb are enriched, and the transport characteristics of the remaining elements show variability in different sand dunes. Mobile dunes have 259.70 ppm Ba, 143.13 ppm Sr, 133.04 ppm Zr, and 143.17 ppm Rb, with 82.20 ppm V, 64.87 ppm Co, 58.94 ppm Ce, 29.37 ppm Cr, 32.65 ppm Ni, 38.13 ppm Zn, and 28.30 ppm Pb; and <20 ppm of Cu, As, Y, Nb, and Ga; V, Co, Ni, As, Pb, Rb, and Ga are enriched; and La, Cr, Cu, Zn, Sr, Ba, Y, Zr, Nb, and Ce are depleted; La, V, Cr, Co, As, Ba and Ce have CV > 18%. Climbing sand sheets are dominated by V, Sr, Ba, Zr, and Rb with an average content of 102.23 ppm, 156.43 ppm, 312.26 ppm, 152.66 ppm, and 107.89 ppm, respectively; the next highest contents of Cr, Co, Zn, Pb, and Ce are 31.00 ppm, 69.15 ppm, 25.23 ppm, 22.32 ppm, and 61.02 ppm, respectively; with average contents of La, Ni, Cu, As, Y, Nb, and Ga < 20 ppm; Cr, Ni, Rb, and Ce are generally consistent with UCC; V, Co, As, and Pb are enriched; and La, Cu, Zn, Sr, Ba, Y, Zr, Nb, and Ga are depleted; La, Cr, Co, Ni, and Ce with CV > 18%. Nebkhas are dominated by Sr, Ba, Zr, and Rb, with average contents of 184.33 ppm, 307.27 ppm, 326.50 ppm, and 144.37 ppm, respectively; the average contents of V, Cr, Co, Ni, Zn, Pb, Y, Ga, and Ce are 90.83 ppm, 32.83 ppm, 29.37 ppm, 25.87 ppm, 41.13 ppm, 26.70 ppm, 29.47 ppm, 21.43 ppm, and 28.90 ppm, respectively, with average contents of La, Cu, As, and Nb < 20 ppm; Cr is generally consistent with UCC, V, Co, Ni, As, Pb, Y, Zr, Rb, and Ga being enriched and La, Cu, Zn, Sr, Ba, Nb, and Ce being depleted; La, Co, Pb, Y, Zr, Rb and Ce have CV > 18%.

### 3.3. Differences in the Geochemical Characteristics of Surface Sediments from Different Types of Sand Dunes

There are differences in elemental content and UCC standardized values for different types of sand dunes (Table 3). The SiO_2_ proportion in mobile dunes and climbing sand sheets differs between 61.67% and 74.22%, whereas in nebkhas it is relatively less variable, varying between 63.82% and 67%. On average, SiO_2_ is higher in climbing sand sheets. The highest concentration of alumina appears in nebkhas, whereas the climbing sand sheets have lower values (av. 7.42%, Table 1). The relatively highest CaO concentration is found in the climbing sand sheets, which is twice as high as in mobile dunes and nebkhas. Compared with UCC (Figure 3), SiO_2_ remains largely consistent with UCC; CaO is enriched in climbing sand sheets and nebkhas and is basically consistent with the UCC in mobile dunes. The rest of the elements have suffered losses to varying degrees. The depletion characteristics of SiO_2_, K_2_O, and Na_2_O are essentially the same for the three dune types compared to the UCC, while the enrichment and depletion characteristics of the remaining major elements are significantly different.

Generally, Ni, Pb, and Nb are present in high concentrations in the mobile dunes, with higher concentrations of V, Cr, Co, Cu, Ba, and Ce in the climbing sand sheets and La, Zn, As, Sr, Y, Zr, Rb, and Ga in the nebkhas (Table 2). The CVs for some trace elements are all >18%, suggesting that trace element composition also shows some different trends for the three types of dunes. Compared to UCC, Ni, Rb, and Ga are enriched in mobile dunes and nebkhas, whereas Ga is depleted, and Ni and Rb are largely consistent with UCC in climbing sand sheets; Y and Zr are enriched in climbing sand sheets and depleted in mobile dunes and nebkhas; Cr is generally consistent with UCC in climbing sand sheets and nebkhas but deficit in mobile dunes; Ce is generally consistent with UCC in climbing sand sheets but deficit in mobile dunes and nebkhas.

## 4. Discussion

### 4.1. Factors Controlling Geochemical Compositions 

Differences in the geochemical composition of aeolian sand should be correlated with sediment provenance, grain size characteristics, chemical weathering, and regional precipitation [39]. An analysis of the PCA in this study showed that 27 elements could be divided into several independent principal components (Table 4). The top five principal components (PCs) with eigenvalues > 1 interpreted 89.98% of the total variance, indicating that these elements showed at least five dominant sources.

At present, PC1 interpreted 45.46% of the total variance, including SiO_2_, Fe_2_O_3_, CaO, MgO, La, MnO, Ni, Zn, As, Pb, TiO_2_, Rb, Nb, and Ga. In these areas, although we cannot currently quantify their roles, the key factors are the grain size characteristic and material source [39,40]. A number of lines of evidence suggest that the geochemical characteristics of the surface sediments are grain-size-dependent [41,42,43,44]. Wind action is one of the main external forces shaping the Earth’s surface morphology, and the sediment grain size is regularly sorted and redistributed by the wind action [40,45], leading to the migration of minerals and geochemical elements, which controls changes in elemental content [42]. On account of the variability of environment and mineral composition, different elements are mainly found in the sand grains of different grain-size levels [46,47,48]. The elements contained in PC1 showed a more significant correlation with the coarse-grained fraction and a better correlation with Mz and σ. Additionally, the other elements also showed a good correlation with the grain size fraction, Mz, and σ (Table 5). Chemical weathering is weak and physical weathering is strong in the Dinggye area, and during the transport process, elements that are resistant to weathering are enriched in the coarse fraction level, while elements that are susceptible to weathering are enriched in the fine fraction [42]. Therefore, PC1 represents the grain size characteristics and is the key factor controlling the major and trace elemental compositions of the different types of dunes.

PC2 interpreted 24.80% of the total variance, including SiO_2_, V, Sr, Ba, Zr, Y, Ce, and P_2_O_5_. Si, V, Sr, Ba, and Y have high loading values in PC2, and they are significantly different in different types of dunes (ANOVA < 0.05), showing that these dune types may differ somewhat in sand sources. Furthermore, there are some differences in the geochemical characteristics of the different types of sand dunes. Therefore, PC2 represents the control role of the provenance. The results suggest that over 80% of the elements in aeolian sands may be due to grain size characteristics and sediment provenance.

In addition, PC 3–5 also explains 9.74%, 5.97%, and 4.02% of the total variance (Table 4), with PC3 including K_2_O and Cu, PC4 including Co, and PC5 including Cr (Table 4). This suggests that there are some other factors, such as chemical weathering and precipitation, that also contribute to the element compositions of the surface deposits. The effect of chemical weathering can influence major oxide concentrations [49]. However, the area is subject to weaker chemical weathering, so relatively minor contributions to elemental content are derived from chemical weathering. Given the relatively small study area, where precipitation differences were not significant, we believe that the effect of precipitation on elemental composition is also relatively small.

### 4.2. Environmental Implications of the Geochemical Characteristics of Different Types of Sand Dunes in the Dinggye Area

**Figure 5:**
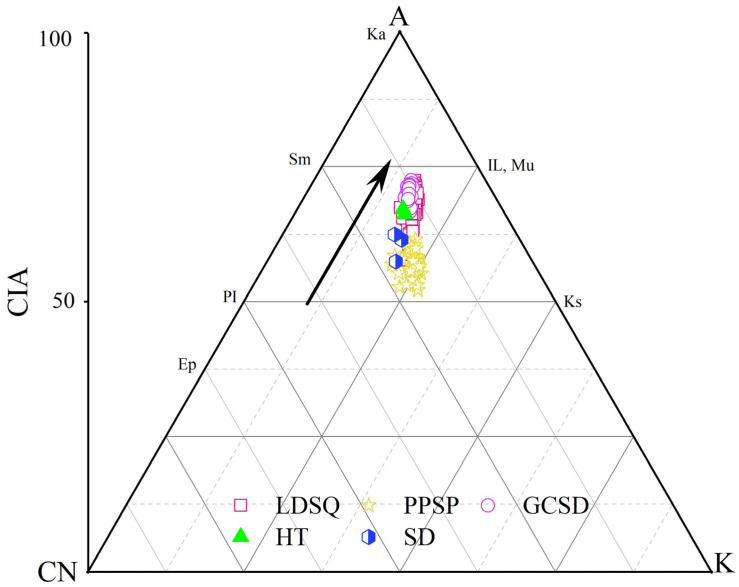
A-CN-K ternary diagram of the modern dune in Dinggye area (the arrows indicate the weathering trend). Ka = Kaolinite; Sm = Smectite; IL = Illite; Mu = Muscovite; Pl = Plagioclase; Ks = K-feldspar, Ep = Epidote; A = Al_2_O_3_; CN = CaO* + Na_2_O; K = K_2_O.

Geochemical characteristics usually reflect the extent of chemical weathering [30,31]. CIA values are a good indicator of the degree of weathering of feldspar into clay minerals, with higher CIA values indicating a greater degree of chemical weathering [30]. CIA values in the range of 50–65 represent a low degree of chemical weathering, reflecting the cold and dry depositional environment [50]. The CIA values for mobile dune, nebkha, and flood plain are similar, with mean values of 66.74, 70.35, and 66.64, respectively (Table 1); the CIA values of climbing sand sheet and lakeshore sediments are similar, with mean values of 56.95 and 60.44, respectively (Table 1). This indicates that the surface sediments in the Dinggye area are in a low chemical weathering stage. A predictive model of continental chemical weathering trends (the A-CN-K diagram) based on mass balance principles, feldspar leaching experiments, and thermodynamic calculations of mineral stability is widely used to reflect chemical weathering trends as well as principal components and mineralogical changes during chemical weathering [30,51]. In the A-CN-K diagram (Figure 5), the triangular projection points of each sample are found above the PI-Ks line; this strongly suggests that the plagioclase is the first to be weathered and decomposed, Na and Ca are depleted, and sand is affected by K-metasomatism.

**Figure 6:**
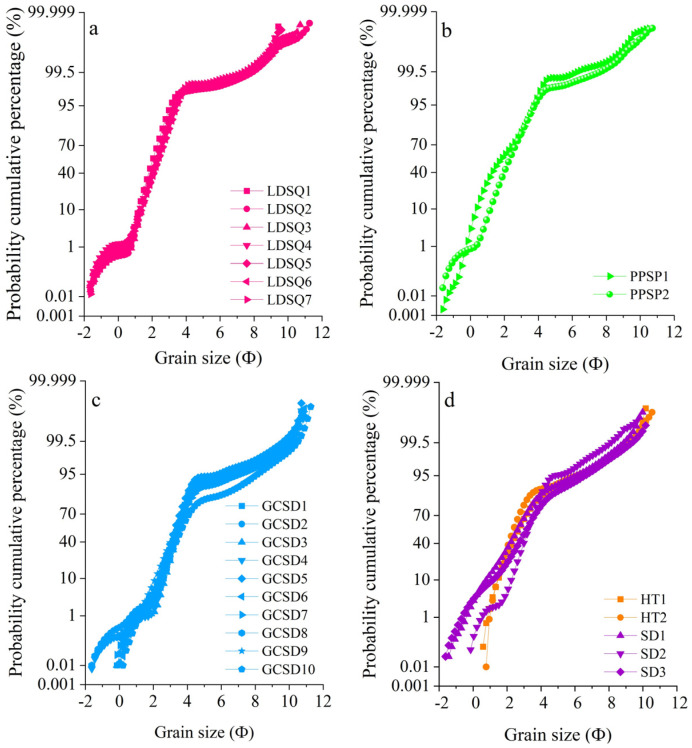
Grain size probability curves in mobile dunes (**a**), climbing sand sheets (**b**), nebkhas (**c**), and flood plain and lakeshore (**d**).

**Figure 7:**
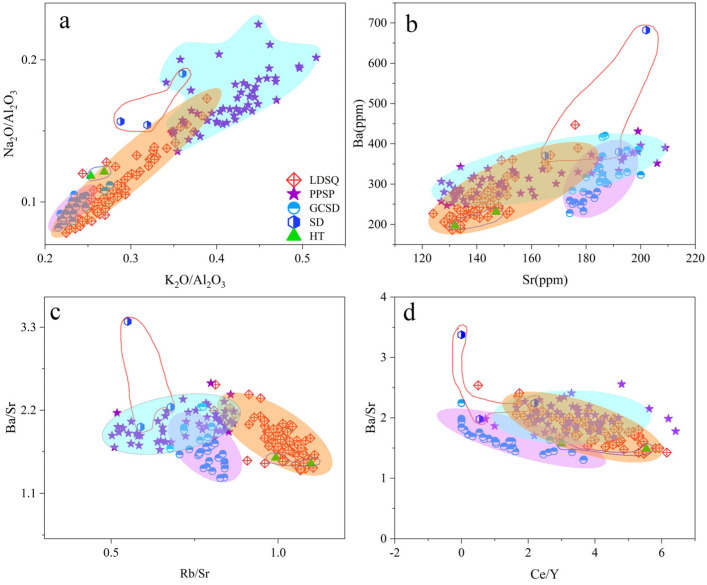
Ratios of the selected elements in the mobile dunes, climbing sand sheets, nebkhas, flood plain, and lakeshore. (**a**) Na_2_O/Al_2_O_3_ vs. K_2_O/Al_2_O_3_ and (**b**) Ba/Sr. (**c**) Ba/Sr vs. Rb/Sr. (**d**) Rb/Sr vs. Ce/Sr.

**Figure 8:**
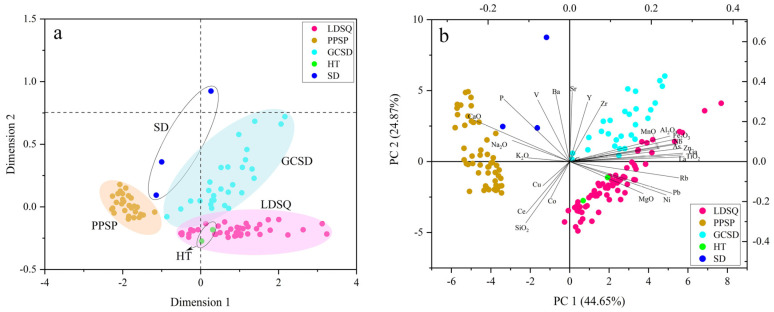
Scatter diagrams of multidimensional scaling analysis (**a**) and principal component analysis (**b**) for mobile dune, nebkha, and flood plain sediments in the Dinggye area.

Dune provenance is an essential topic in the study of aeolian landforms, with their source characteristics and the dynamics that drive dune formation dominating the formation and evolution of dunes [52]. The probabilistic accumulation curves for both mobile dunes and nebkhas are three-stage (Figure 6a,b), with the transport modes mainly being reptation, saltation, and suspension, of which the saltation component accounts for the largest proportion. Climbing sand sheets have two and three stages, with saltation being the dominant transport mode (Figure 6c). The samples of lakeshore and flood plain are predominantly two-stage (Figure 6d). The mode of transport of aeolian sediments in the Dinggye area is predominantly saltation, suggesting that the sediments are mainly transported over short distances and that the provenance is mainly near-source. The geochemical characteristics of aeolian sand can be used to determine the sediment provenance [42,47,52,53]. In the Ba/Sr scatter plot (Figure 7b), the aeolian sediments of each dune tend to be dispersed and separated, indicating that they may have a single source and may originate in situ on a local scale. The ratios of Na_2_O/Al_2_O_3_ to K_2_O/Al_2_O_3_, Ba/Sr to Rb/Sr, and Ba/Sr to Ce/Y in the sands from the mobile dune, nebkha, and flood plain sediments are similar (Figure 7a,c,d), suggesting that mobile dune and nebkha sediments are derived from a similar provenance, and that flood plain sediments are likely to be their source. The climbing sand sheet and the lakeshore exhibit similar Na_2_O/Al_2_O_3_ versus K_2_O/Al_2_O_3_, Ba/Sr versus Rb/Sr, and Ba/Sr versus Ce/Y ratios (Figure 7a,c,d), which indicate that their geochemical compositions are similar and that the lakeshore is a significant provenance for the climbing sand sheet. Recent studies have often used MDS and PCA to trace the provenance of aeolian sediments, and they have demonstrated their applicability [6,7,52,53]. We selected 39, 27, 27, 2, and 3 samples each from mobile dune, climbing sand sheet, nebkha, flood plain, and lakeshore, respectively, and we performed an MDS analysis of their major and trace elements (Figure 8a). PCA was performed using the major and trace elements of all samples (Figure 8b). In Figure 8a, all three types of dunes can be separated, the mobile dunes and nebkhas are closer, and the samples of flood plain are close to them; the samples of climbing sand sheet and lakeshore are close to each other. In Figure 8b, the samples of the climbing sand sheet are different from those of the mobile dune and nebkha; the samples of the flood plain are similar to the mobile dune and nebkha, and the climbing sand sheet is similar to the lakeshore. As a matter of fact, the MDS and PCA methods proved to be more effective than the elemental ratio biplots [4,6,7,15]. The MDS and PCA plots indicate that the flood plain is the sand source for the mobile dune and nebkha and that the lakeshore is the sand source for the climbing sand sheet. Each summer, the water levels of the river and lake rise, with the river and lake water being closer to the dunes, providing sufficient coarse sand particles. When the dunes contain more water, the dune sand is not easily carried away by wind power; in winter, the river and lake level drop, and the dunes are farther away from the river and are subject to strong southwest wind deflation, transport, and accumulation [27,29]. The prevailing wind direction in the region is SSW, with a continuous monsoon for eight months on average annually. The topography of the Dinggye area is high in the west and low in the east, and the winds are stronger in winter due to the control of cold high pressure [29]. The course of the river valley is almost parallel to the wind direction, which strengthens the wind, and the superposition with the valley wind makes the southwest wind stronger [2]. Intense wind action is a major factor for aeolian activity in this region [24,25]. The prevailing local wind conditions suggest sand transport from the floodplain and lakeshore to the dunes. In summary, the geochemical characteristics indicate that the flood plain and lakeshore are important sand sources in the Dinggye area. The sand provenance analysis in this region makes an important contribution to the understanding of the distribution of sand dunes; meanwhile, it can enrich the theory of aeolian landforms in China and also provide a scientific basis for the control of land desertification in the Dinggye area.

## 5. Conclusions

A detailed analysis of grain size and major and trace elements of different sand types in the Dinggye area has led to the following conclusions.

(1) The major elements are mainly SiO_2_, Al_2_O_3_, and CaO; SiO_2_ is essentially the same as UCC, and CaO is enriched; the trace elements are mainly Ba, Sr, Zr, and Rb; V, Co, As, and Pb are enriched. Mobile dunes, climbing sand sheets, and nebkhas have differences in chemical element composition and are UCC-normalized. MgO, TiO_2_, Ni, Pb, and Nb are present in high concentrations in mobile dunes, with higher concentrations of SiO_2_, CaO, K_2_O, Na_2_O, P_2_O_5_, V, Cr, Co, Cu, Ba, and Ce in climbing sand sheets; and higher Al_2_O_3_, Fe_2_O_3_, La, Zn, As, Sr, Y, Zr, Rb, and Ga in nebkhas. Compared with UCC, CaO is more enriched in climbing sand sheets, Ni, Rb, and Ga are enriched in mobile dunes and nebkhas; and Y and Zr are enriched in nebkhas.
(2) An analysis of PCA showed that the five principal components explained 89.98% of the total variance. PC1 explained 45.46% of the total variance, and the elements were more significantly correlated with the coarse-grained fraction and better correlated with Mz and σ. Thus, PC1 represents the grain size characteristics and is the key factor controlling the major and trace element composition of the different types of dunes. PC2 explained 24.80% of the total variance, representing the sand source. PC3–5 also explained 9.74%, 5.97%, and 4.02% of the total variance, indicating that other factors such as chemical weathering and precipitation also influenced the elemental composition of the surface sediments.
(3) Geochemical characteristics can reflect chemical weathering and provenance information. The CIA and A−CN−K triangles indicate that the different dune types are in a lower chemical weathering stage, with plagioclase weathering and decomposition first, and then Na and Ca depletion. The transport mode of aeolian sediment in the Dinggye area is mainly saltation, indicating that the sediment is mainly transported for short distances and that the provenance is mainly near the source. The combination of grain size characteristics, elemental ratios, MDS, PCA, and geomorphological conditions suggests that the flood plain is the sand source of the mobile dunes and nebkhas and that the lakeshore is the provenance of the climbing sand sheet.

## Figures and Tables

**Table 1:** Average content of major elements in aeolian sediments (%). *n* indicates the number of samples.

Major Elements	Samples									
	Mobile Dunes (*n* = 71)		Climbing Sand Sheets (*n* = 53)		Nebkhas (*n* = 30)		Flood Plain (*n* = 2)		Lakeshore (*n* = 3)	
	Mean	CV	Mean	CV	Mean	CV	Mean	CV	Mean	CV
SiO_2_	68.63	4.03	69.14	3.69	65.29	1.41	67.08	0.52	66.90	3.57
Al_2_O_3_	10.64	14.62	7.42	11.82	12.39	5.22	11.79	1.25	8.87	9.78
CaO	4.06	15.45	11.47	24.40	5.60	10.15	3.66	5.22	11.58	19.03
Fe_2_O_3_	2.36	15.69	1.71	7.08	2.40	7.19	2.24	10.73	2.11	8.78
MgO	1.06	13.55	0.69	28.17	1.03	9.68	1.27	4.45	1.87	22.25
K_2_O	2.95	3.97	3.05	3.95	2.88	3.63	3.11	2.96	2.84	3.52
Na_2_O	1.14	7.99	1.26	9.30	1.17	5.13	1.43	0.50	1.47	2.97
TiO_2_	0.24	22.62	0.10	14.86	0.23	16.69	0.20	5.08	0.16	24.81
MnO	0.03	16.07	0.02	5.60	0.03	5.47	0.03	3.85	0.03	9.68
P_2_O_5_	0.08	17.12	0.14	17.61	0.12	9.88	0.07	5.38	0.16	20.73
CIA	66.74	5.43	56.95	4.02	70.35	1.99	66.64	1.01	60.44	4.38

**Table 2:** Average content of trace elements in modern dunes (ppm).

Trace Elements	Samples									
	Mobile Dunes (*n* = 71)		Climbing Sand Sheets (*n* = 53)		Nebkhas (*n* = 30)		Flood Plain (*n* = 2)		Lakeshore (*n* = 3)	
	Mean	VC	Mean	VC	Mean	VC	Mean	VC	Mean	VC
La	18.06	27.50	3.81	56.98	18.13	19.84	14.00	10.10	10.67	53.31
V	82.20	21.22	102.23	17.74	90.83	7.58	68.50	5.16	116.00	15.59
Cr	29.37	55.11	31.00	38.26	32.83	17.03	87.00	813	44.67	50.87
Co	64.87	56.15	69.15	43.98	29.37	29.60	86.50	22.07	68.33	87.03
Ni	32.65	12.84	17.75	26.28	25.87	9.51	28.00	5.05	15.67	35.15
Cu	10.77	7.56	10.81	8.12	9.33	14.71	10.00	0	11.00	15.75
Zn	38.13	14.00	25.23	5.80	41.13	9.33	36.00	11.79	31.67	14.92
As	10.04	23.29	5.77	15.43	10.60	9.47	8.00	17.68	7.00	14.29
Sr	143.13	7.20	156.43	16.32	184.33	3.78	139.50	7.60	186.33	10.27
Pb	28.30	3.98	22.32	6.07	26.70	20.04	28.50	2.48	21.67	5.33
Ba	259.70	19.73	312.26	13.58	307.27	16.54	214.00	11.23	477.33	37.15
Y	15.62	14.61	19.30	16.21	29.47	27.85	14.50	14.63	34.00	36.85
Zr	133.04	17.16	152.66	17.72	326.50	38.90	121.00	21.04	346.67	47.65
Rb	143.17	2.16	107.89	3.59	144.37	26.20	145.50	0.49	112.00	0.89
Nb	11.97	17.35	8.26	5.89	12.37	11.92	10.50	6.73	10.67	14.32
Ga	19.90	6.42	14.23	5.45	21.43	5.15	19.50	3.62	15.67	3.69
Ce	58.94	26.02	61.02	23.24	28.90	80.85	60.00	28.28	32.50	58.74

**Table 3:** Differences and similarities in the geochemical characteristics of different types of sand dunes.

Dunes	Differences	Similarities
Mobile dunes	MgO, TiO_2_, Ni, Pb, and Nb are higher; Ni, Rb, and Ga are enriched	SiO_2_ and CaO are enriched in major elements and other elements are depleted; V, Co, As, and Pb are enriched in trace elements
Climbing sand sheets	SiO_2_, CaO, K_2_O, Na_2_O, P_2_O_5_, V, Cr, Co, Cu, Ba, and Ce are higher; Cr, Ni, Rb, and Ce are consistent with UCC	
Nebkhas	Al_2_O_3_, Fe_2_O_3_, La, Zn, As, Sr, Y, Zr, Rb, and Ga are higher; V, Co, Y, Zr, Ni, Rb, and Ga are enriched; Cr is consistent with UCC	

**Table 4:** Principal components (PCs) loading values for the contents of elements/oxides in aeolian sediments in the Dinggye area.

Element/Oxide	Component				
	PC1	PC2	PC3	PC4	PC5
SiO_2_	−0.384	−0.813	−0.282	0.153	0.134
Al_2_O_3_	0.858	0.351	0.115	−0.207	−0.156
Fe_2_O_3_	0.903	0.284	0.291	0.008	0.069
CaO	−0.814	0.537	0.185	0.067	0.008
MgO	0.653	−0.505	−0.384	0.026	−0.075
K_2_O	−0.385	0.135	0.503	0.464	−0.092
Na_2_O	−0.521	0.252	−0.142	0.413	0.141
La	0.977	0.041	0.060	0.036	0.106
V	−0.262	0.805	0.462	0.137	−0.052
Cr	0.088	0.102	−0.061	−0.438	0.810
MnO	0.772	0.146	0.471	0.147	0.259
Co	−0.173	−0.487	−0.144	0.782	0.194
Ni	0.822	−0.428	0.089	0.292	−0.017
Cu	−0.229	−0.311	0.783	−0.226	−0.132
Zn	0.959	0.175	0.042	0.135	−0.005
As	0.897	0.240	0.207	0.098	0.040
Sr	0.042	0.898	−0.047	−0.036	−0.289
Pb	0.874	−0.400	−0.038	0.197	−0.045
Ba	−0.100	0.885	0.087	0.187	0.197
TiO_2_	0.962	0.075	0.133	0.003	0.100
Y	0.194	0.742	−0.547	0.040	−0.015
Zr	0.305	0.672	−0.606	−0.041	−0.068
Rb	0.932	−0.202	−0.050	−0.058	−0.187
Nb	0.920	0.208	0.069	0.195	0.123
Ga	0.964	0.120	0.016	−0.033	−0.135
Ce	−0.407	−0.637	0.403	−0.154	−0.053
P_2_O_5_	−0.561	0.811	0.035	0.048	−0.021
%variance	45.456	24.795	9.737	5.973	4.023
Cumulative % of total variance explained	45.456	70.251	79.988	85.961	89.984

Extraction method: principal component analysis. Five components extracted.

**Table 5:** Correlation analysis of grain size fractions and parameters with elements.

Elements	Coarse Sand	Medium Sand	Fine Sand	Very Fine Sand	Silt	Clay	Mz	σ
SiO_2_	−0.59 **	0.397 **	0.396 **	−0.437 **	−0.469 **	−0.179 **	−0.326 **	−0.250 **
Al_2_O_3_	−0.513 **	−0.333 **	0.099	0.415 **	0.458 **	0.408 **	0.551 **	−0.244 **
Fe_2_O_3_	−0.502 **	−0.027	0.180 **	0.151	0.254 **	0.246 **	0.338 **	−0.372 **
CaO	0.879 **	−0.048	−0.685 **	−0.039	−0.067	−0.363 **	−0.400 **	0.723 **
MgO	−0.790 **	−0.101	0.597 **	0.157	0.124	0.364 **	0.440 **	−0.616 **
K_2_O	0.474 **	0.297 **	−0.214 **	−0.345 **	−0.261 **	−0.369 **	−0.473 **	0.253 **
Na_2_O	0.513 **	−0.050	−0.355 **	−0.047	−0.037	−0.132	−0.238 **	0.419 **
La	−0.717 **	−0.045	0.379 **	0.172 *	0.231 **	0.317 **	0.438 **	−0.0553 **
V	0.625 **	−0.040	−0.643 **	0.039	0.104	−0.232 **	−0.222 **	0.552 **
Cr	−0.136	0.024	−0.057	0.081	0.125	0.002	0.118	0.013
MnO	−0.404 **	0.310 **	0.254 **	−0.169 *	−0.026	0.029	0.037	−0.445 **
Co	−0.022	0.404 **	0.257 **	−0.400 **	−0.368 **	−0.216	−0.320 **	−0.210 *
Ni	−0.732 **	0.241 **	0.643 **	−0.154	−0.074	0.205 **	0.184 *	−0.712 **
Cu	0.149	0.449 **	0.232 **	−0.514 **	−0.460 **	−0.283 **	−0.459 **	−0.074
Zn	−0.635 **	−0.162 *	0.231 **	0.289 **	0.358 **	0.359 **	0.495 **	−0.418 **
As	−0.473 **	−0.019	0.134	0.159	0.252 **	0.240 **	0.325 **	−0.388 **
Sr	0.351 **	−0.643 **	−0.688 **	0.613 **	0.601 **	0.241 **	0.356 **	0.615 **
Pb	−0.780 **	0.094	0.645 **	−0.033	0.029	0.328 **	0.308 **	−0.699 **
Ba	0.427 **	−0.192 *	−0.612 **	0.243 **	0.257 **	−0.136	0.006	0.473 **
TiO_2_	−0.659 **	0.006	0.372 **	0.117	0.183 *	0.275 **	0.366 **	−0.547 **
Y	−0.019	−0.669 **	−0.545 **	0.765 **	0.717 **	0.280 **	0.600 **	0.367 **
Zr	0.134	0.705 **	−0.446 **	0.796 **	0.734 **	0.367 **	0.675 **	0.265 **
Rb	−0.767 **	−0.134	0.488 **	0.196 *	0.250 **	0.455 **	0.491 **	−0.550 **
Nb	−0.582 **	−0.069	0.226 **	0.207 *	0.273 **	0.273 **	0.403 **	−0.433 **
Ga	−0.660 **	−0.259 **	−0.273 **	0.349 **	0.384 **	0.434 **	0.551 **	−0.421 **
Ce	0.162 *	0.489 **	0.311 **	−0.592 **	−0.554 **	−0.232 **	−0.519 **	−0.136
P_2_O_5_	0.708 **	−0.339 **	−0.803 **	0.307 **	0.277 **	−0.157	−0.056	0.778 **

**. At the 0.01 level (two-tailed), the correlation is significant. *. At the 0.05 level (two-tailed), the correlation is significant.
